# Special Issue “Emerging Diseases/Viruses: Prevention, Control, Surveillance, and One Health”

**DOI:** 10.3390/tropicalmed7100301

**Published:** 2022-10-15

**Authors:** Yannick Simonin

**Affiliations:** Pathogenesis and Control of Chronic and Emerging Infections, University of Montpellier, INSERM, Etablissemnt Français du Sang, 34000 Montpellier, France; yannick.simonin@umontpellier.fr

Zoonotic diseases account for at least 60% of all infectious diseases and no less than two-thirds of new emerging ones, which underlines the importance of monitoring them as early as possible. Understanding the nature of the animal-to-human transmission of zoonotic diseases is a fundamental requirement for their effective anticipation and control. The SARS-CoV-2 pandemic, and more recently, the monkeypox epidemic, have revealed our limited preparedness against a diversity of emerging and re-emerging pathogens. There are different modes of transmission of zoonotic diseases: direct or indirect contacts, vector-borne, or environmental (water, soil, food, etc.), making their monitoring more complex. Effective control of zoonotic diseases requires early detection of the source of the disease and the factors that contribute to its spread. Combining wildlife, farm animal, and domestic animal health monitoring with human health monitoring can greatly reduce the risk of major epidemics or pandemics of zoonotic origin.

The changes in our environment caused mainly by human activity and the evolution of human–animal interactions are and will undoubtedly be responsible for several new health crises. These crises will be manifested in particular by an increase in the frequency and intensity of epidemics and epizootics. Numerous factors favor the increase in interactions between humans, animals, and their environment, such as the increase in animal and human population movements, or the demographic increase in the human population and its expansion into new geographical areas (ultimately increasing human/wildlife interactions). Over half of known human pathogenic diseases can be aggravated by climate change, including warming, precipitation, and floods [1]. These profound changes can have major consequences for human health. Consequently, the need to understand the emergence of a disease in humans through an approach that integrates a large number of environmental parameters, described as global, has been reinforced.

The links and analogies between animal health and human health have been known since Antiquity, particularly with the transposition of anatomical knowledge from animals (particularly from dissections) to humans. This notion was expressed more concretely in the mid-1800s by a Prussian pathologist, Rudolf Virchow, who emphasized the lack of distinction between animal and human medicine. The “One Health” concept has since developed by basing the study of these issues on multidisciplinary and multisectoral approaches [2]. Its general principle is to study the interactions between animals, humans, and their various environments. Despite its rich content and the fact that the concept has been around for a long time, its practical application remains very limited and is confronted with organizational difficulties. It is in the fight against zoonoses, which are responsible for emerging diseases that tend to become epidemic or even endemic, that the One Health concept was applied the earliest and most effectively. Indeed, awareness of the importance of this concept became more prevalent during health crises initially involving animal health, particularly in Africa. There are many examples of surveillance of zoonotic diseases using integrated approaches, particularly in areas of the world where humans, domestic animals, and wildlife live in close contact. Examples include brucellosis in many countries, including the Middle East; bovine tuberculosis in sub-Saharan Africa; leptospirosis in Fiji; and Ebola in West Africa. These examples underline the need for integrated approaches to the efficient management of complex health problems. The COVID-19 crisis has demonstrated the need to better implement integrated approaches to health and has prompted us to look ahead to the post-crisis period and to anticipate the management of future crises. Historically focused on zoonotic issues, the One Health concept has evolved into a broader disciplinary field, including food safety, water safety, biodiversity, and climate change adaptation. This evolution is due to the awareness that the factors at the origin of health problems are multiple and complex and are not limited to the study of the direct or indirect interactions between animals and humans.

In this Special Issue, we invite colleagues to submit original research articles and scientific reviews to assemble a collection of papers highlighting the progress in our understanding of all aspects related to surveillance and control of zoonotic diseases, including (1) the development of new diagnostic tools, (2) outbreak investigation and surveillance programs of emerging pathogens (including One Health approaches), and (3) understanding the mechanisms of pathogen emergence. The first 10 papers published in this Special Issue address topics related to viruses (SARS-CoV-2, influenza, DENV, WNV, ZIKV), bacteria (*Escherichia coli*), and parasites (Leishmania, Plasmodium) [3,4,5,6,7,8,9,10].
